# Evaluation of lyophilized bacteriophage cocktail efficiency against multidrug-resistant *Salmonella* in broiler chickens

**DOI:** 10.1186/s12866-024-03467-2

**Published:** 2024-09-11

**Authors:** Nehal M. Nabil, Maram M. Tawakol, Abdelhafez Samir, Heba M. Hassan, Mona Mohieldin Elsayed

**Affiliations:** 1 Reference Laboratory for Veterinary Quality Control on Poultry Production, Animal Health Research Institute (AHRI), Agricultural Research Center (ARC), Nadi El-Seid Street, Dokki, Giza 12618 Egypt; 2 Department of Hygiene and Zoonoses, Faculty of Veterinary Medicine, Mansoura University, Mansoura, 35516 Egypt

**Keywords:** Biocontrol, Phage stability, Poultry farms, *S.* Kentucky

## Abstract

Currently, phage biocontrol is increasingly used as a green and natural technology for treating *Salmonella* and other infections, but phages exhibit instability and activity loss during storage. Therefore, in this study, the effects of lyophilization on the activity and stability of phage cocktails for the control of multidrug-resistant *Salmonella* in broiler chickens were determined. Eight serotypes of *Salmonella* were isolated and identified from broiler chicken farms, and bacteriophages against multidrug-resistant *Salmonella* enterica subsp. enterica serovar Kentucky, *Salmonella* enterica subsp. enterica serovar Typhimrium and *Salmonella* enterica subsp. enterica serovar Enteritidis were isolated. The bacteriophage cocktail was prepared and lyophilized, and it was subjected to in vitro and in vivo examinations. A reconstituted lyophilized bacteriophage cocktail was used for the oral treatment of chicks before and after challenge with multidrug-resistant *S.* Kentucky. The colonization of cecum by *S.* Kentucky was detected by using real-time PCR, and the serum levels of IgM, IgA and IL-4 and pathological changes in the different groups were detected. Three Caudovirales phages families were identified including *Autographiviridae*, *Straboviridae* and *Drexlerviridae* against multidrug-resistant *S.* Kentucky, *S.* Typhimrium *and S.* Enteritidis. The groups treated with the bacteriophage cocktail showed no clinical signs, no postmortem lesions, and a mortality rate of 0%, which improved the growth performance parameters. Additionally, the estimated serum levels of IgM, IgA and IL-4 were significantly greater in the bacteriophage cocktail-treated groups. Lyophilization effectively preserves the long-term storage stability of phages. Therefore, lyophilized bacteriophage cocktail therapy is a valuable approach for controlling multidrug-resistant *Salmonella* infections in broiler chickens.

**Supplementary Information:**

The online version contains supplementary material available at 10.1186/s12866-024-03467-2.

## Introduction

Avian salmonellosis is a global concern in the poultry industry [1]. It is a serious disease that impedes the development of the poultry industry, particularly in developing countries [2]. *Salmonella* is one of the major causes of foodborne outbreaks worldwide and is transmitted to humans from infected poultry [3]. producing gastroenteritis [4]. Poultry acts as the main reservoir for various nontyphoidal *Salmonella* (NTS) strains, including *S.* Typhimurium and *S*. Enteritidis serotypes, between food-producing animals [5], and *S.* Enteritidis *and S*. Typhimurium are responsible for 34% and 17.5%, respectively, of poultry-related foodborne illnesses [6].

Antibiotic resistance has become a major public health issue after decades of widespread use. The increase in the prevalence of multidrug-resistant *Salmonella* serotypes in the food chain is having a wide global impact [4]. Antibiotic side effects raise doubts about the efficacy of some antimicrobial therapies, necessitating the development of biocontrol strategies [7]. Biocontrol refers to the use of one or more organisms such as bacteriophages to inhibit other organisms. Bacteriophages are abundantly present naturaly in every environment suitable for bacterial growth and are already present in all the foods we consume. Bacteriophage is considered as a green technology because it is ecofriendly product to the environment, non-toxic and can be readily used at multiple points in food processing without sacrificing the quality or safety of the product [8].

Bacteriophages are viruses that kill bacterial cells [8] through invasion and propagation, resulting in bacterial lysis [9]. Phage therapy is emerging as an alternative strategy for controlling multidrug-resistant *Salmonella* in young chicks [10]. In comparison to those of antibiotics, the great success rate and safety of phage therapy are attributed in part to its specificity for selected bacteria and its ability to invade only one species, serotype, or strain without destroying the commensal bacterial flora [11]. It can also modulate the microbiome of the chicken intestinal tract, reduce pathogenic microbial populations and allow the development of beneficial microbiota [12], which help enhance gut morphology [9]. Phages lose their activity with long-term storage and with exposure to high temperatures, pH, and ionic strength [13]. Lyophilization is a well-established technology for bacteriophage storage [14] that **is** used in the preparation of pharmaceutical products to improve their stability under physical and chemical stresses for long-term storage. Lyophilization with suitable excipients such as sucrose, gelatin or sucrose is beneficial for phages [15].

### Aim of study

This research aimed to evaluate the ability of a lyophilized cocktail of bacteriophages as an advanced alternative strategy to control multidrug-resistant *Salmonella* infection in broiler chickens.

## Materials and methods

### Sample collection

This study was carried out in accordance with the guidelines of the Guide for the Care and Use of Laboratory Animals and approved by the Medical Research Ethics Committee of Mansoura University with code number MU-ACUC (VM.R.23.08.118).

One hundred diseased broiler chickens (aged 30 to 38 days) were selected from 10 broiler poultry farms (10 birds from each farm) in Dakahlia Governorate, Egypt. The farms were selected according to the willingness of the owners to permit sample collection. All the selected diseased birds were subjected to clinical and *postmortem* (PM) examinations because they suffered from watery diarrhea, poor growth, weakness, ruffled feathers and enteritis. Samples from internal organs (liver, heart, cecum and spleen) were collected individually according to [16] and then pooled together as one sample for each bird. The samples were transported immediately for the isolation of multidrug-resistant *Salmonella* strains in the Reference Laboratory for Veterinary Quality Control on Poultry Production (RLQP).

### Multidrug-resistant *Salmonella* strains

*Salmonella* strains were isolated and identified from the collected internal organs according to previous methods [17]. Briefly, 1 gm of each sample was added to 9 ml of nutrient broth (Oxoid, UK) and incubated at 37 °C for 18 h for pre-enrichment. Then, 1 ml of each broth was transferred to 9 ml of selenite F broth (Oxoid, UK) and incubated at 37 °C for 18 h. A loopful of incubated selenite F broth was streaked into XLD (Oxoid, UK) plates and incubated at 37 °C for 18 h. The plates were checked for the growth of typical colonies of *Salmonella spp*. Biochemical testes for identification, including the urase test, triple sugar iron (TSI), lysine decarboxylase test, indole test, citrate utilization test and sucrose, xylose, rhamnose and arabinose fermentation tests. According to the Kauffman-White scheme [18], the serological identification of *Salmonella* isolates for the detection of somatic and flagellar antigens was performed in the Reference Laboratory for Veterinary Quality Control on Poultry Production (RLQP).

According to the guidelines of the Clinical and Laboratory Standards Institute [19], the in vitro susceptibility of all confirmed *Salmonella* isolates was examined using the disc diffusion method on Mueller–Hinton agar (Oxoid, UK). Antimicrobial agent selection was based on the importance of antimicrobial agents in both the human and veterinary fields in addition to their antimicrobial mechanisms. Ten antibiotics belonging to seven classes (from Oxoid, UK) were selected: oxytetracycline (OT, 30 µg), doxycycline (DO, 30 µg), colistin sulfate (CT, 25 µg), streptomycin (S, 10 µg), neomycin (N, 30 µg), ampicillin/sulbactam (SAM, 20 µg), amoxicillin (AMX, 10 µg), sulfamethoxazole-trimethoprim (SXT, 25 µg), norfloxacin (NOR, 10 µg), and florfenicol (FFC, 30 µg). *S.* Typhimurium (ATCC 14028) was used as a positive control.

### Bacteriophage isolation and purification

Five sewage samples were collected from different poultry farms for the isolation of bacteriophages against 3 selected serotypes of multidrug-resistant *Salmonella* including *S.* Enteritidis, *S.* Typhimurium and *S.* Kentucky [20]. The 3 selected serovars were the most common causes of foodborne illness worldwide. Ten milliliters of each sample was added to 90 mL of phosphate-buffered saline (PBS) in a sterile Whirl-Pak bag with a filter screen. The mixture was centrifuged (10,000 rpm) for 10 min to remove the fecal debris and then filtered with pore syringe filters (0.22 μm). The filtrate for each sample was used for phage isolation via the double-layer plate method. In brief, 300 µL of each of the previously prepared serovars which contain 3 × 10^7^ CFU/mL approximately was mixed with 100 µL of the sample filtrate and 4 mL of soft agar (tryptone soya broth containing 0.6% agar; Oxoid Ltd., Basingstoke, UK). Then, the mixture was spread onto a freshly prepared tryptone soya agar plate (containing 1.5% agar; Sigma‒Aldrich, St. Louis, USA). This method was performed independently with each of the 3 serovars for each filtrate. Overlay plates were incubated for 24 h at 30 °C, followed by phage purification [19] to obtain supernatant samples containing viable phages.

The prepared bacteriophages were tested via the spot technique for 3 individual serovars, namely, *S.* Kentucky, *S.* Typhimurium and *S.* Enteritidis. Two hundred microliters of the target bacterial suspension were incubated overnight, spread on LA plates and incubated for 40 min at 30 °C. Ten microliters of individual phage lysates were spotted onto the surface of the plates at a titer of 10^9^ phages/ml. The plates were left to dry and were inspected for lysis zones after an overnight incubation at 30 °C. A spot assay was used to assess the bactericidal ability of the isolated virulent phages to form clear zones on the bacterial strains and was repeated three times for each phage [21].

A plaque assay was performed by tenfold serial dilution of 0.1 ml of the obtained phage suspension. A single colony of *S.* Kentucky, *S.* Typhimurium or *S.* Enteritidis cultured overnight was inoculated into 5 ml of buffered peptone water (each liter contains 5 gm of yeast extract) and incubated for 3 h at 37 °C with agitation. 3 ml of 0.5% semisolid agar were placed in a water bath at 45 °C, and then 0.1 ml of phage and 0.5 ml each of *S.* Kentucky, *S.* Typhimrium and *S.* Enteritidis were added to semisolid agar tubes, gently mixed and poured onto nutrient agar plates, which were incubated overnight at 37 °C. The obtained plaques were detected and counted as plaque forming units (PFUs) [20]. A single plaque was subjected to bacteriophage purification and propagation [22]. In brief, a single plaque was picked and put into 5.0 ml of buffered peptone water containing 100 µl of bacterial host and then incubated at 37 °C under shaking at 1200 rpm. The phage host mixture was centrifuged at 6000 rpm for 10 min, and the supernatant was filtered with 0.22 μm pore size filter to exclude any bacterial contamination.

### Morphological characterization of the isolated phages

The morphological characteristics of the purified bacteriophage particles were examined by transmission electron microscopy (JEOL-2100, Japan), and the particles were classified according to Zhu et al. [23]. The samples were stained using uranyl acetate, a drop of each suspension which contain 10^10^ PFU/ml was placed on 200 mesh copper grids with carbon-coated Formvar films, and the excess was discarded using filter paper. A saturated solution of uranyl acetate was placed on the grids, and the excess was discarded and examined via electron microscopy [22].

The bacteriophage cocktail solution was prepared according to the results of electron microscopy by mixing an equal ratio (1:1:1) of the recorded phages in this study at an equal concentration (10^10^ PFU/ml) [24].

### Lyophilization of the bacteriophage cocktail

The lyophilization of the bacteriophage cocktail was performed according to Manohar and Ramesh [15] with some modifications as follows: 2 ml of solution was prepared using 1500 µl of 1% gelatin and 500 µl of bacteriophage cocktail stock solution (10^10^ PFU/ml) in 10 ml lyophilization bottles with stoppers and lyophilized using a BIOBASE freeze dryer (MODEL; BK-FD10PT). Gelatin form polymers that support the maintenance of phage morphology during the process of lyophilization. In brief, the lyophilization cycles were conducted as follows: the sample holding shelves were cooled to 5 °C, and the samples were precooled to − 20 °C. After the samples were loaded, the vials containing the shelves were cooled to − 30 °C (1 °C/minute) and maintained for 90 min. The complete solidification of the cocktail solution was ensured within 90 min at − 30 °C. Primary drying was maintained at − 30 °C for 12 h at 100 °C. During the secondary drying process, the temperature was increased from − 30 °C to 25 °C (0.1 °C/minute) for 10 hours at 100 °C. After the lyophilization process, the vials were sealed and stored at 4 °C with 0% relative humidity.

### Stability and viability of the lyophilized bacteriophage cocktail

The stability and viability of the lyophilized bacteriophage were checked [25]. The vials were resuspended in 1 ml of sterile 0.9% NaCl solution as a rehydrating solution. The resuspended solution was serially diluted and then tested for bacteriophage enumerations using a plaque assay technique at which the number of plaques represents viable bacteriophages. The active bacteriophage concentration before and after lyophilization was detected using a plaque assay technique to determine the decrease in bacteriophage activity during the lyophilization process. The resuspended solution was also examined for bacteriophage morphology using transmission electron microscopy as previously described. A bacteriophage cocktail solution was used as a control.

### In vivo efficiency of a lyophilized bacteriophage cocktail against multidrug-resistant *S.* Kentucky

#### Experimental chicks

Fifty specific pathogen-free (SPF) chicks at one-day-old with a weight of 40–41 g for each chick. The chicks were provided with feed and water *ad libitum* and maintained at an age-appropriate temperature throughout the experimental period. The experimental chicks were kept in separate cages at biosecurity two animal facilities at the Animal Health Research Institute, Giza, Egypt.

#### Bacterial challenge strains

Multidrug-resistant *S.* Kentucky was selected from this study and used for challenge purposes on the basis of showing multidrug- resistance to the most of antimicrobial agents commonly used in broiler farms and showing good results with the in vitro preparation of individuals and groups of bacteriophages (cocktails).

#### Application of the lyophilized bacteriophage cocktail

The lyophilized vial was resuspended in 1 ml of sterile 0.9% NaCl solution. The resuspended solution was added to one liter of sterile drinking water, and the sterility of the solution was checked by streaking onto a nutrient agar (Oxoid, UK.) The plates were incubated for 24 h at 37 °C.

### Experimental design

The chicks were confirmed to be *Salmonella* free by cloacal swabbing and plated on specific agar plates [16]. The experimental chicks were randomly divided into 5 groups, each group contained 10 chicks. Each group was marked and placed individually. The groups were as follows: the 1st group was not challenged and not treated (negative control), the 2nd group was challenged without treatment (positive control), the 3rd group was not challenged, the 4th group was treated before challenge, and the 5th group was treated after challenge. In the 2nd, 4th and 5th groups, the challenge was performed with *S.* Kentucky on the 2nd day of age at a dose of 10^5^ CFU/ml orally according to Nabil et al. [26]. Moreover, in the 3rd, 4th and 5th groups, each chick was treated with a resuspended lyophilized bacteriophage cocktail at 1, 2, 3, 6, 8, and 10 days of age. The experiment ended at 15 days of age (13 days post challenge; 13 dpc).

### Observation of chicks

The health status of the experimental chicks was observed twice daily during the experimental period. Clinical signs and mortality rates were recorded for each group until the end of the experiment. Growth performance parameters such as initial and final body weight (BW) (at 1st day and 15th day of age), body weight gain (BWG), feed intake (FI) and the feed conversion ratio (FCR) were calculated and reported at the end of the experimental period. FI and FCR calculation were conducted according to Gabriel et al. [27].

### Bacteriological examinations

The spontaneously dead chicks were necropsied and subjected to PM examinations, and samples from the liver, spleen and cecum were collected aseptically and subjected to *S*. Kentucky isolation and identification. At the end of the experiment, the chicks were humanely euthanized via cervical dislocation, necropsied and subjected to PM examination in addition to the collection of cecum, liver and spleen samples, after which they were subjected to *S.* Kentucky isolation. To count *S.* Kentucky bacteriologically, one gram of the cecal contents was homogenized, serially diluted and then plated onto XLD agar. The plates were incubated at 37 °C for 24 h, after which the CFUs of *S.* Kentucky were counted (CFU/gm). The representative colonies of *S.* Kentucky were confirmed by slide agglutination with poly (O and H) and serotype-specific antisera.

### Bacteriophage count in the cecal samples of experimental chicks

Bacteriophages were counted in the cecal contents in groups 3, 4 and 5 [28]. One gram of cecal content was homogenized in 10 mM MgSO4 buffer and then subjected to serial dilutions. The dilutions were plated onto a lawn of *S.* Kentucky and incubated at 37 °C for 24 h as described above for the plaque assay. The bacteriophage plaques were counted (PFU/g).

### Real-time PCR technique for *Salmonella* quantification in the cecal samples

Real-time PCR is an accurate quantitative and rapid method for *Salmonella* DNA quantification. The real-time PCR was used in the current study to confirm the results of bacteriological count from groups 1, 2, 4 and 5 to evaluate *S.* Kentucky colonization (CFU per gram of ceca). DNA was extracted using a QIAamp DNA Mini Kit (Qiagen, Germany, GmbH) with modifications according to the manufacturer’s instructions. The oligonucleotide primers and probes were obtained from Metabion (Germany) (Supplementary Table 1). DNA amplification was performed in a final volume of 25 µl containing 3 µl of DNA template, 12.5 µl of 2x QuantiTect Probe real-time PCR Master Mix, 8.875 µl of PCR grade water, 0.25 µl of each primer (50 pmol conc.) and 0.125 µl of each probe (30 pmol conc.). Primary denaturation was performed for 15 min at 94 °C, followed by 40 cycles of denaturation at 94 °C for 15 s, annealing at 49 °C for 30 s and extension at 72 °C for 10 s. The reactions were performed in a Stratagene real-time PCR machine (MX3005P).

### Immunological assays

Serum samples were collected from the blood of the experimental chicks (*n* = 3 for each group). Immunoglobulin (IgM) was measured using chicken IgM enzyme-linked immunosorbent assay (ELISA) kits (EAGEL, BIOSCIENCES). An Abcam IgA chicken ELISA kit (Abcam^®^) was used for the estimation of IgA. A chicken interleukin 4 (IL-4) ELISA kit was also used for the measurement of IL-4 (ABBEXA). All the examinations were performed according to the manufacturer’s instructions.

### Histopathological examinations

Liver and cecum tissue samples were collected from 3 chicks for each group for histopathological examinations. Tissue samples were prepared through the following stages: Fixation, processing, embedding and sectioning according to Alturkistani et al. [29]. The prepared samples were stained with hematoxylin and eosin [30].

### Statistical analysis

The statistical analysis was conducted using SPSS version 20. The P value was calculated by one-way ANOVA (Bonferroni test) to detect significant differences between the experimental groups. A P value of 0.05 was considered to indicate statistical significance.

## Results

### *Salmonella* isolation, serotyping and antimicrobial sensitivity

A total of 18 Salmonella isolates were recovered from 100 diseased broiler chickens (18%). Eight Salmonella serotypes were recorded: 5 of *S.* Kentucky, 4 of *S.* Typhimrium, 3 of *S.* Enteritidis, 2 of *S.* Infantis and 1 of each *S.* Virchow, *S.* Rechovot, *S.* Papuana, and *S.* Labadi. The most predominant serotype was *S.* Kentucky. The disc diffusion tests revealed resistance to amoxicillin at 94.4%, ampicillin/sulbactam at 83.3%, oxytetracycline at 72.2%, colistin sulfate at 66.7%, streptomycin at 66.7%, florfenicol at 50%, sulfamethoxazole-trimethoprim at 44.4%, doxycycline at 44.4%, neomycin at 38.9% and norfloxacin at 27.8% (Table 1). The majority of the isolated Salmonella strains were multidrug- resistant. 83.3% of Salmonella isolates (15 out of 18) were multidrug- resistant to more than 3 or more antimicrobial classes, with a multidrug-resistance index (MDRI) ranging from 0.4 to 0.9 (Table 2).

**Table 1:** Antimicrobial resistance of *Salmonella* isolates

Serotype (no.)	OT	CT	*N*	S	AMX	SAM	SXT	NOR	FFC	DO
*S*. Enteritidis (3)	2	1	1	0	3	1	1	1	2	1
*S*. Infantis (2)	2	2	1	2	2	2	0	2	2	2
*S.* kentucky (5)	3	3	3	2	5	5	3	1	2	3
*S*. Labadi (1)	1	0	0	1	1	1	0	1	1	0
*S*. Papuana (1)	0	1	1	1	1	1	1	0	0	0
*S*. Rechovot (1)	0	1	0	1	1	1	1	0	1	1
*S*.Typhimrium (4)	4	3	1	4	3	4	2	0	1	1
*S*. Virchow (1)	1	1	0	1	1	0	0	0	0	0
Total (18)	13	12	7	12	17	15	8	5	9	8
Susceptible (%)	27.8%	33.3%	61.1%	33.3%	5.6%	16.7%	55.6%	72.2%	50%	55.6%
Resistant (%)	72.2%	66.7%	38.9%	66.7%	94.4%	83.3%	44.4%	27.8%	50%	44.4%

*OT* oxytetracycline, *CT* colistin sulphate, *N* neomycin, *S* streptomycin, *AMX* amoxicillin, *SAM* ampicillin/sulbactam, *SXT* sulfamethoxazole- trimethoprim, *NOR* norfloxacin, *FFC* florfenicol, *DO* doxycycline

**Table 2:** Antimicrobial resistant pattern profiles of Salmonella isolates

Antimicrobial agent pattern profiles	Antimicrobial agent	NO. of isolates	No. of resistance markers	MDRI
1	CT, N, S, AMX, SAM, SXT	1	6	0.6
2	CT, S, AMX, SAM, SXT, FFC, DO	1	7	0.7
3	OT, CT, AMX, SAM, NOR, FFC	1	6	0.6
4	OT, CT, N, AMX, SAM, SXT, NOR, DO	1	8	0.8
5	OT, CT, N, S, AMX, SAM, FFC, DO	1	8	0.8
6	OT, CT, N, S, AMX, SAM, NOR, FFC, DO	1	9	0.9
7	OT, CT, N, S, AMX, SAM, SXT, FFC, DO	1	9	0.9
8	OT, CT, S, AMX	1	4	0.4
9	OT, CT, S, AMX, FFC	1	5	0.5
10	OT, CT, S, AMX, SAM, NOR, FFC, DO	1	8	0.8
11	OT, CT, S, AMX, SAM, SXT	1	6	0.6
12	OT, CT, S, AMX, SAM	1	5	0.5
13	OT, N, AMX, SXT, FFC, DO	1	6	0.6
14	OT, N, S, AMX, SAM, SXT, DO	1	7	0.7
15	OT, S, AMX, SAM, NOR, FFC	1	6	0.6

*OT* oxytetracycline, *CT* colistin sulphate, *N* neomycin, *S* streptomycin, *AMX* amoxicillin, *SAM* ampicillin/sulbactam, *SXT* sulfamethoxazole- trimethoprim, *NOR* norfloxacin, *FFC* florfenicol, *DO* doxycycline

### Bacteriophage isolation and morphological characterization

**Fig. 1:**
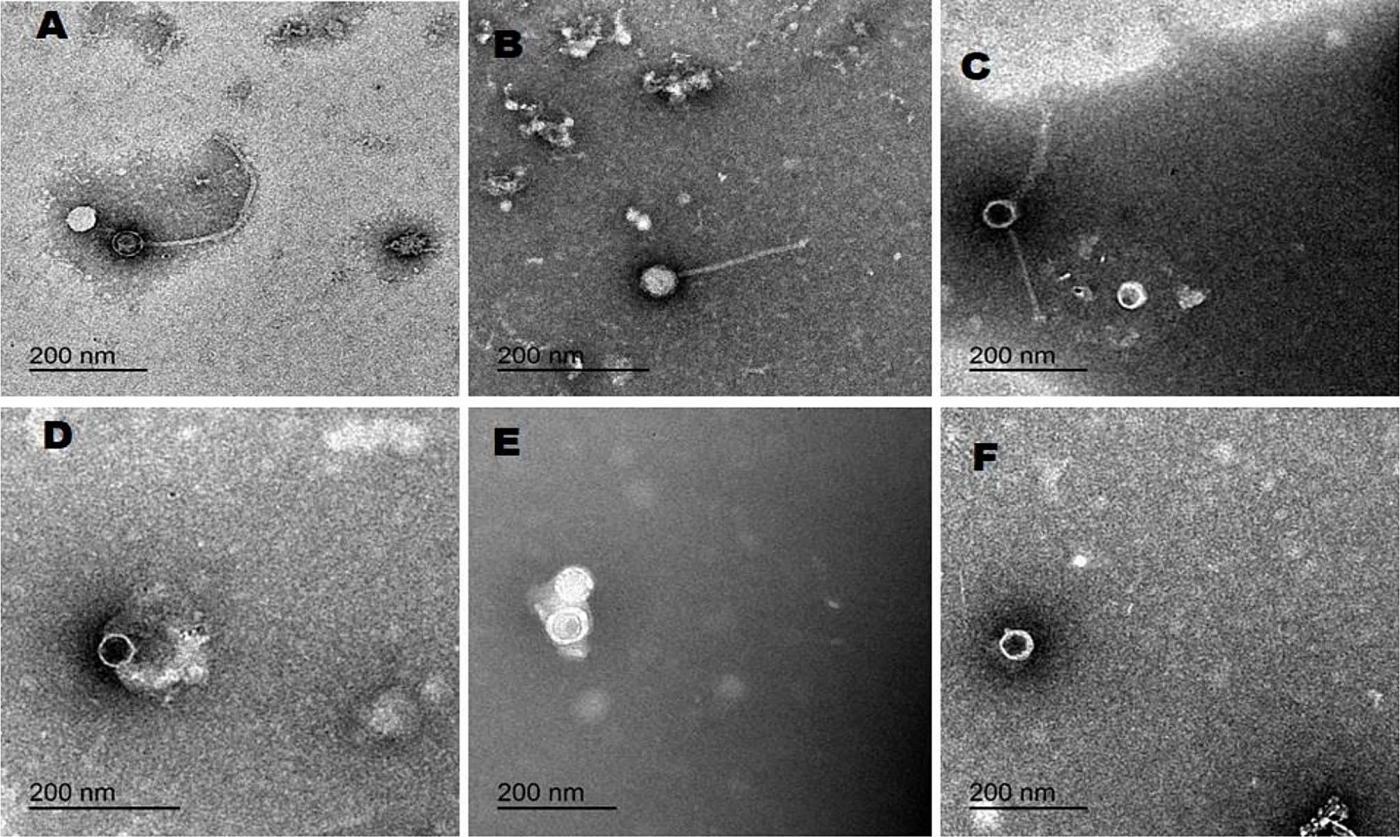
Transmission electron microscopy photograph of isolated bacteriophages. **A** & **B**: belong to *Siphoviridae* family, **C**: is belonging to *Myoviridae* family. **D**, **E** & **F**: belong to *Podoviridae* family

Different specific and lytic bacteriophages for MDR *Salmonella* were recorded from the collected sewage samples and confirmed using spot and plaque techniques. Among the 5 collected swage samples, 3 exhibited phage activity against *S.* Kentucky, *S.* Typhimrium and *S.* Enteritidis. Three different plaques with different plaque diameters were picked and selected for purification and propagation. The bacteriophages examined by electron microscopy (Fig. 1) revealed 3 different Caudovirales phages that were represented into 3 families according to their morphological characteristics: noncontractile long tail which belonging to *Autographiviridae* family, contractile tail which belonging to *Straboviridae* family and short tail and without tail which belonging to *Drexlerviridae* family.

### Stability and viability of the lyophilized bacteriophage cocktail

**Fig. 2:**
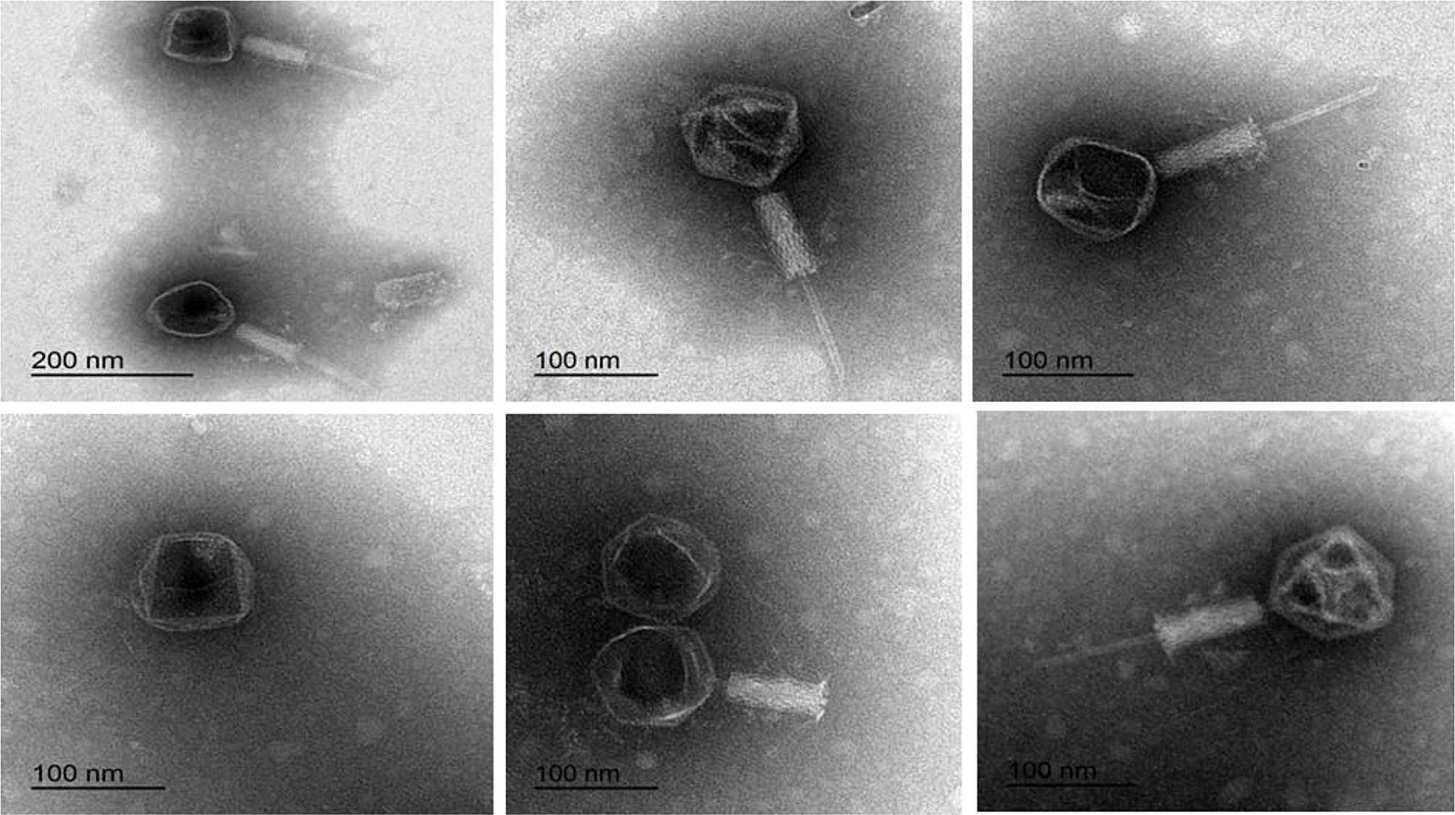
Transmission electron microscopy photograph of isolated bacteriophages after lyophilization and rehydration

After rehydration with sterile 0.9% NaCl solution, the stability, viability and morphology of the lyophilized bacteriophage cocktail were evaluated via electron microscopy, and the results showed the stability and viability of the bacteriophages in terms of the production of lytic zones via plaque assays. The active bacteriophage concentrations before and after lyophilization were 1 × 10^10^ and 9 × 10^9^ PFU/ml, respectively. The morphological characterization by electron microscopy showed no changes from that recorded before lyophilization (Fig. 2).

### Efficacy of a lyophilized bacteriophage cocktail against multidrug-resistant *S.* Kentucky in experimental chicks

**Fig. 3:**
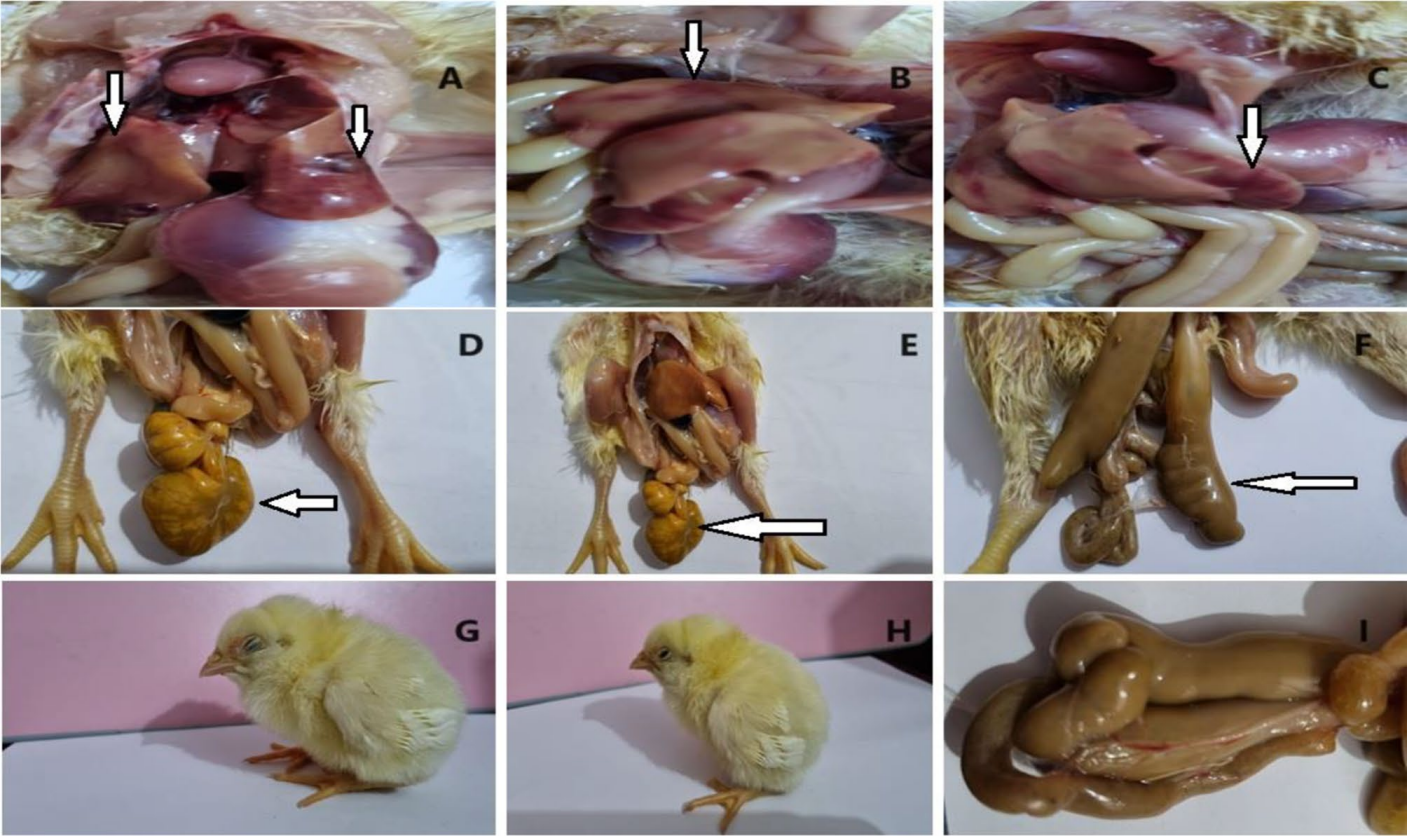
Clinical signs and postmortem changes of the experimental chicks in 2nd group (positive control) which challenged with *S*. Kentucky. **A**, **B** and **C**: congested liver with petechial hemorrhages. **D** and **E**: unabsorbed and enlarged yolk sac. **F** and **I**: enlarged cecum filled with diarrhea. **G** and **H**: chicks showed reluctant to move with closed eyes

Clinical signs, including loss of appetite, poor growth, a decreased weight of the wing, reluctance to move, closed eyes and watery diarrhea with venting, were observed in the 2nd group at 3 days post challenge and persisted during the experimental period (Fig. 3). Groups 1, 3 and 4 showed no clinical signs until the end of the experiment. Chicks in group 5 showed mild diarrhea from 4 dpc.

The recorded mortalities were 0% (0/10) in all groups except for the 2nd group. Additionally, the PM lesions in group 2 included enlarged and congested livers, enlarged spleens, enteritis, congested internal organs, unabsorbed yolk sacs and caeca that were enlarged and filled with diarrhea (Fig. 3). However, in group 5, chicks showed slight enlargement of the cecum and a congested liver. Groups 1, 3 and 4 showed no PM lesions. There was a significant difference in feed intake between the 1st, 2nd and 3rd groups. Additionally, there was a significant difference between the 5th group and the 2nd group in terms of body weight gain and final body weight (Table 3).

**Table 3:** Growth performance parameters of the experimental chicks (mean ± standard error)

Groups Items	Group 1	Group 2	Group 3	Group 4	Group 5
Initial BW (g)	40.2 ± 0.133	40.4 ± 0.163	40.4 ± 0.163	40.4 ± 0.163	40.1 ± 0.1
Final BW (g)	542.1 ± 0.482	286.1 ± 4.642	546.6 ± 0.452	545.9 ± 0.233	535.5 ± 0.224
BWG (g)	502.1 ± 0.504	245.7 ± 4.697	506.2 ± 0.467	505.5 ± 0.269	495.4 ± 0.267
FCR (g)	1.2 ± 0.001	1.53 ± 0.04	1.19 ± 0.002	1.19 ± 0.001	1.2 ± 0.001
FI (g)	601.5 ± 0.342	373.7 ± 3.99	601.5 ± 0.4	600.2 ± 0.3	595.1 ± 0.348

### Recovery and colonization of *S.* Kentucky

After necropsy, *S.* Kentucky was isolated and identified from the cecum, liver and spleen. By plating on XLD agar, chicks in groups 1, 3 and 4 were negative for S. Kentucky colonization. Chicks in groups 2 and 5 were positive for *S.* Kentucky. All of the representative colonies of S. Kentucky were confirmed using serotype-specific antisera, and all of them were positive.

By plating on XLD agar, the mean CFU of *S.* Kentucky in the cecum was detected only in groups 2 and 5, with 1.6 × 10^5^ CFU/gm and 3.1 × 10^1^ CFU/gm, respectively. Groups 1, 3 and 4 were negative for *S*. Kentucky.

**Fig. 4:**
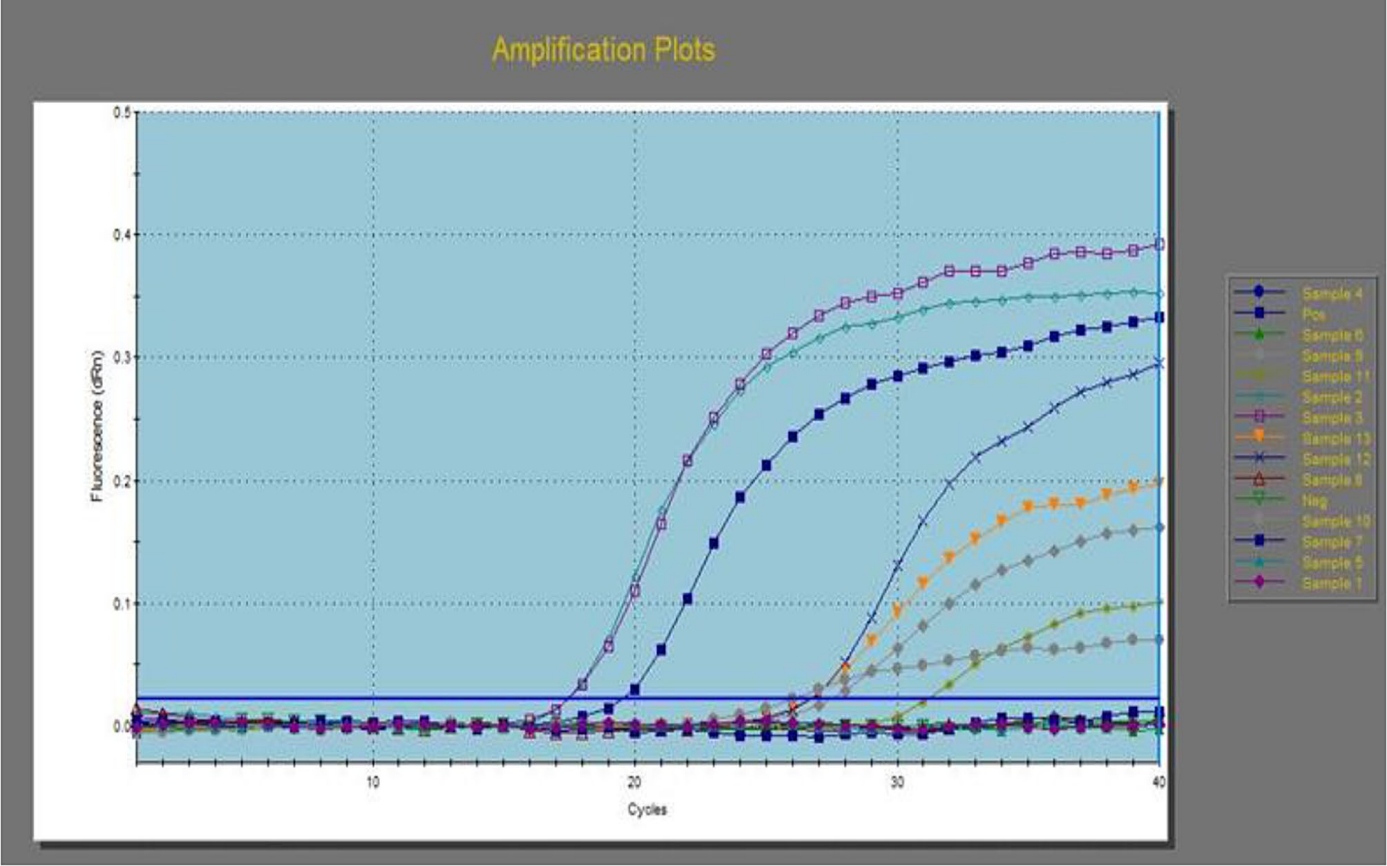
Amplification plot of RT- PCR for *S*. Kentucky colonization in the examined cecal samples. *S*. Kentucky not detected in the cecal samples of the 1st group (samples) and 4th group (samples ). Meanwhile, cecal samples in 2nd group (samples) and 5th group (samples) showed *S*. Kentucky colonization with 1.160 × 10^5^ to 1.554 × 10^5^ and 1.407 × 10^1^ to 5.068 × 10^2^, respectively

Quantitative real-time PCR was used as a rapid and accurate technique to determine *S.* Kentucky loads in caecum of necropsied chicks in the five groups to investigate the significance and the effectiveness of bacteriophage treatments on *S.* Kentucky colonization. Table. 4 and Fig. 4 revealed the absence of *S*. Kentucky cecal colonization in groups 1 and 4. However, cecal samples in group 2 (positive control) and group 5 showed *S*. Kentucky colonization of 1.160 × 10^5^ to 1.554 × 10^5^ and 1.407 × 10^1^ to 5.068 × 10^2^, respectively.

**Table 4:** Colonization of *S. Kentucky* (CFU/ gm) in the cecum of the chicks under experiment

Sample no.	Group. no.	Results	CT.	Concentration (CFU/g)
1	1	Negative	-	-
2	2	+	17.53	1.554 × 10^5^
3		+	17.96	1.160 × 10^5^
4	4	Negative	-	-
5		Negative	-	-
6		Negative	-	-
7		Negative	-	-
8		Negative	-	-
9	5	Positive	27.71	1.531 × 10^2^
10		Positive	25.95	5.068 × 10^2^
11		Positive	31.22	1.407 × 10^1^
12		Positive	26.38	3.783 × 10^2^
13		Positive	26.19	4.305 × 10^2^

### Bacteriophage count in the cecal samples of experimental chicks

A plaque assay revealed that the mean counts of bacteriophages in the cecal contents (PFU/gm) of groups 3, 4 and 5 were 8.6 × 10^7^, 4.39 × 10^8^ and 1.69 × 10^8^ PFU/gm, respectively.

### Immunological assays

**Fig. 5:**
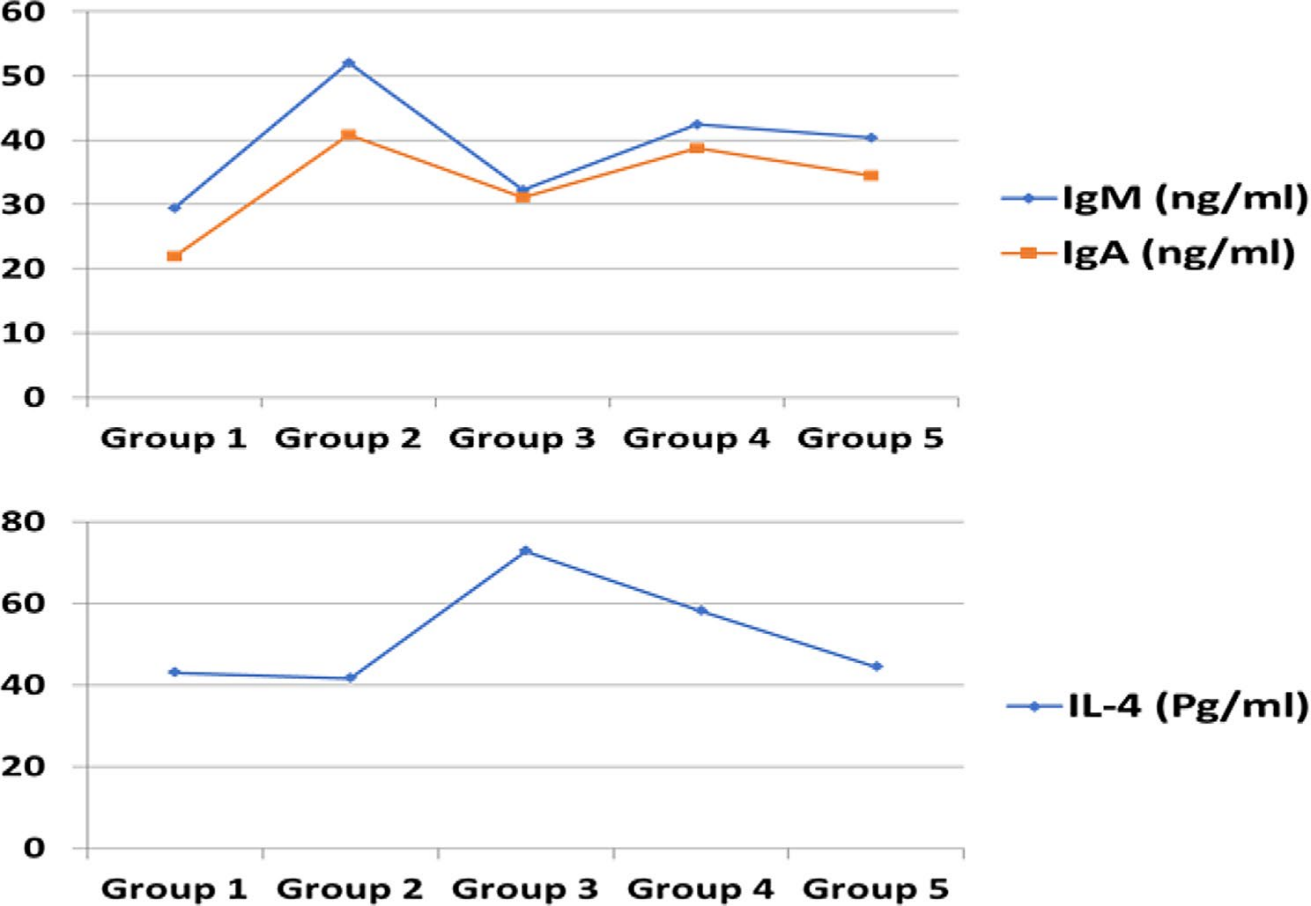
The curve of mean with standard error (mean ± SE) for IgM (ng/ml), IgA (ng/ml) and IL-4 (Pg/ml) results in the different 5 groups. 1st group: negative control, 2nd group: challenged with *S*. Kentucky without treatment, 3rd group: treated with bacteriophages cocktail without challenge, group 4: treated with bacteriophage before the challenge, 5th group: treated after the challenge

The levels of interleukin 4 (IL-4) were significantly greater in groups 3, 4 and 5, which received the bacteriophage cocktail, than in the control groups including groups 1 and 2 (Tables 5 and Fig. 5). Moreover, the level of IL-4 in group 3 (treated with bacteriophage and not challenged with *S.* Kentucky) was greater than that in groups 4 and 5 (treated with bacteriophage and infected with S. Kentucky).

**Table 5:** Immunological response of experimental chicks in different groups (mean ± SE)

Group no. Parameters	Group 1	Group 2	Group 3	Group 4	Group 5
IgM (ng/ml)	29.5 ± 0.8	52 ± 0.55	32.33 ± 0.48	42.4 ± 0.54	40.37 ± 0.44
IgA (ng/ml)	21.87 ± 0.69	40.83 ± 0.67	31.17 ± 0.79	38.83 ± 0.84	34.47 ± 0.58
IL-4 (pg/ml)	42.97 ± 0.62	41.77 ± 0.60	72.93 ± 0.38	58.13 ± 0.75	44.53 ± 0.50

The measured serum levels of IgM and IgA antibodies in response to S. Kentucky challenge were significantly greater in groups 2, 3, 4 and 5 than in the negative control group (group 1). Furthermore, there was a greater percentage of bacteria in groups 3, 4 and 5, which received bacteriophage cocktail treatments, than in control group 1 (Tables 5 and Fig. 5).

### Histopathological examinations

**Fig. 6:**
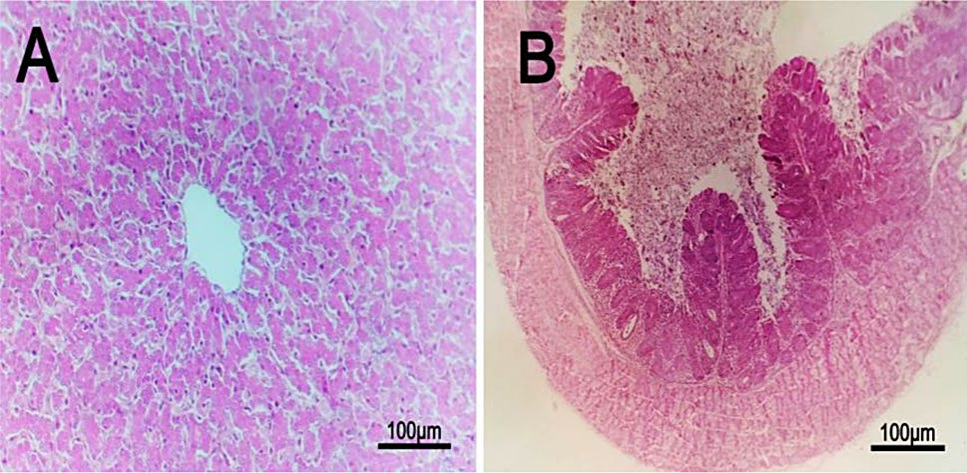
Histopathological lesions in liver and cecal tissue for the negative control chicks (1st group; no challenge and no treatment) with H & E (hematoxylin and eosin), 100×. **A**: Liver with normal tissue architecture and cellular details. **B**: Cecum with normal mucosa, muscularis, submucosa and serosa

**Fig. 7:**
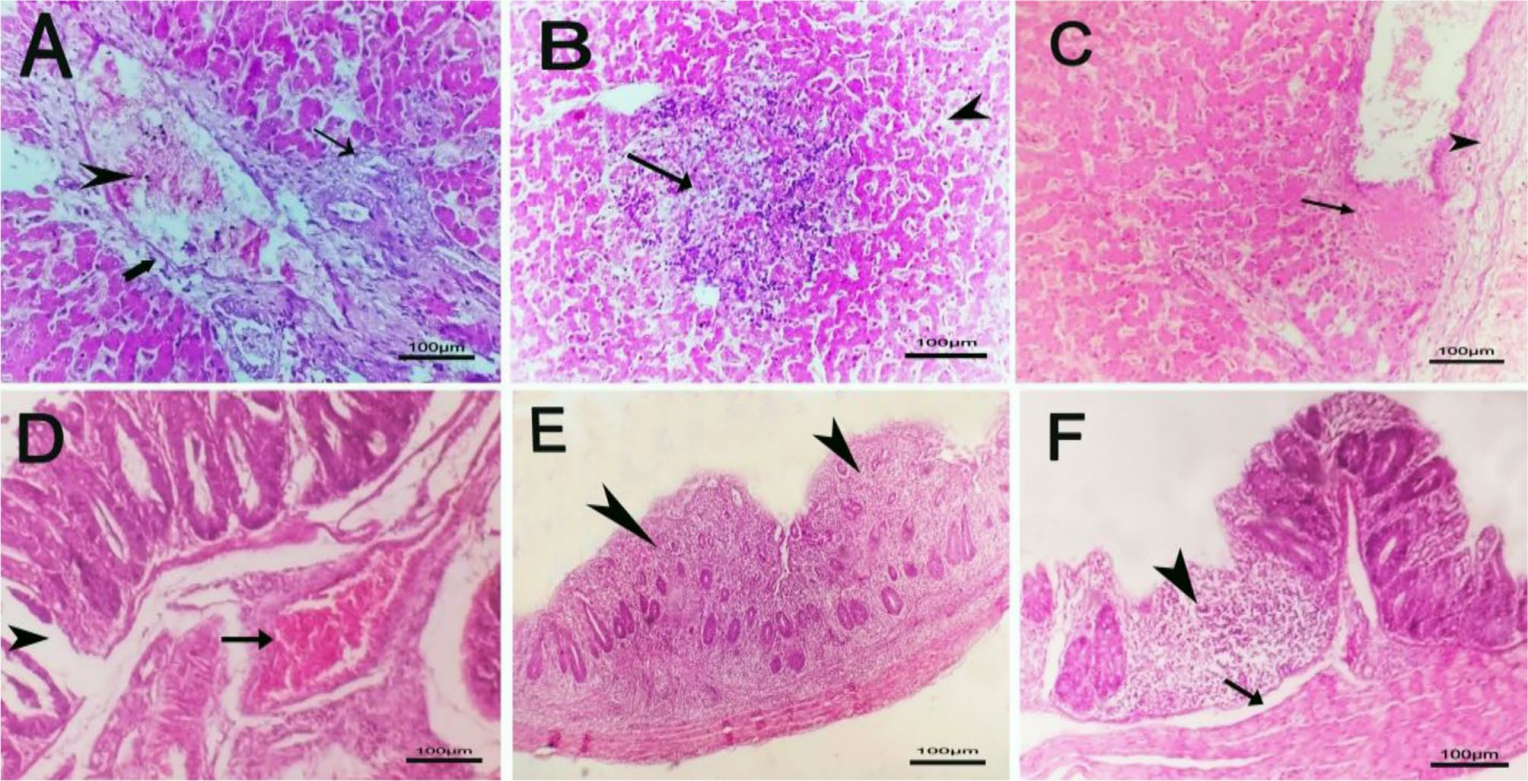
Histopathological lesions in liver and cecal tissue for the challenged chicks without treatment (2nd group) with H&E (hematoxylin and eosin), 100×. **A**: liver with perivascular fibrosis and edema (thick arrow) with severe congestion of hepatic blood vessels (arrowhead) and proliferation of bile ductules (thin arrow). **B**: Liver with focal area of inflammatory mononuclear cells infiltration (arrow) and mild sinusoidal dilation (arrowhead). **C**: Liver with focal necrotic area (arrow) with perivascular fibrosis (arrowhead). **D**: Cecum with severe congestion of submucosal blood vessel (arrow). **E**: Cecum with fusion of some intestinal villi (arrowheads). **F**: caecum with edema beneath submucosa (arrow) with focal distortion and aplasia of submucosal glands(arrowhead)

**Fig. 8:**
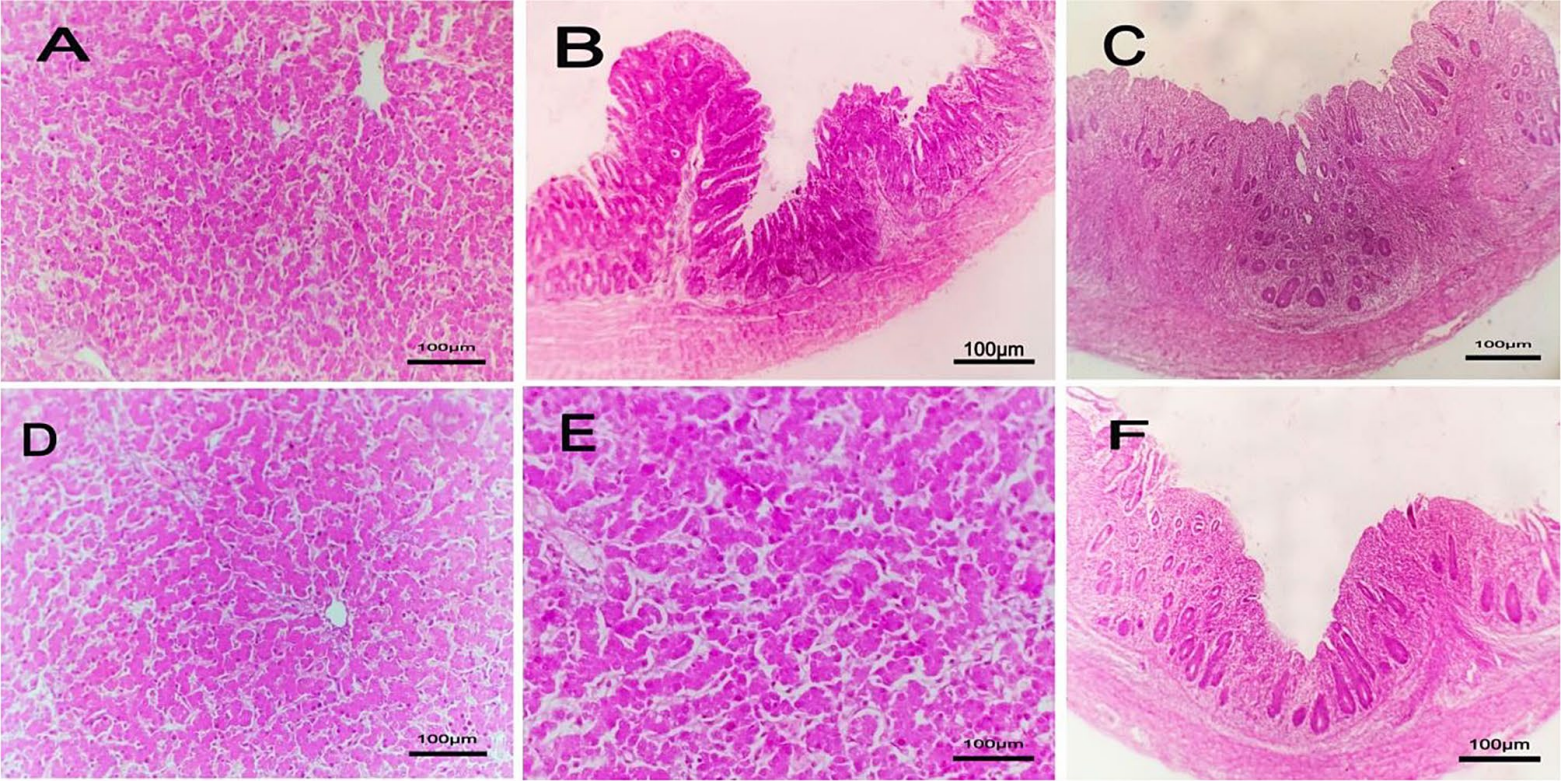
Histopathological lesions in liver and cecal tissue for the 3^rd*^ group which was treated without challenging and 4^th**^ group which was treated before challenging with H & E (hematoxylin and eosin), 100×. A*, D** and E**: liver with normal tissue architecture and cellular details. B*, C* and F**: Cecum with normal mucosa, muscularis, submucosa and serosa

The microscopic findings in group 1 (negative group), group 3 (treated with bacteriophage cocktail) and group 4 (treated with bacteriophage cocktail and then challenged with S. Kentucky and treated with cocktail) revealed normal tissue architecture and cellular details of the liver. Additionally, the cecum showed normal mucosa, muscularis, submucosa and serosa (Figs. 6 and 7). The positive control group (2) showed a focal necrotic area with perivascular fibrosis in the liver, edema with severe congestion of hepatic blood vessels and proliferation of bile ductules in addition to a focal area of inflammatory mononuclear cell infiltration and mild sinusoidal dilation. The cecum of group (2) showed severe congestion of submucosal blood vessels`, fusion of some intestinal villi, edema beneath the submucosa with focal distortion and aplasia of the submucosal glands (Fig. 8).

**Fig. 9:**
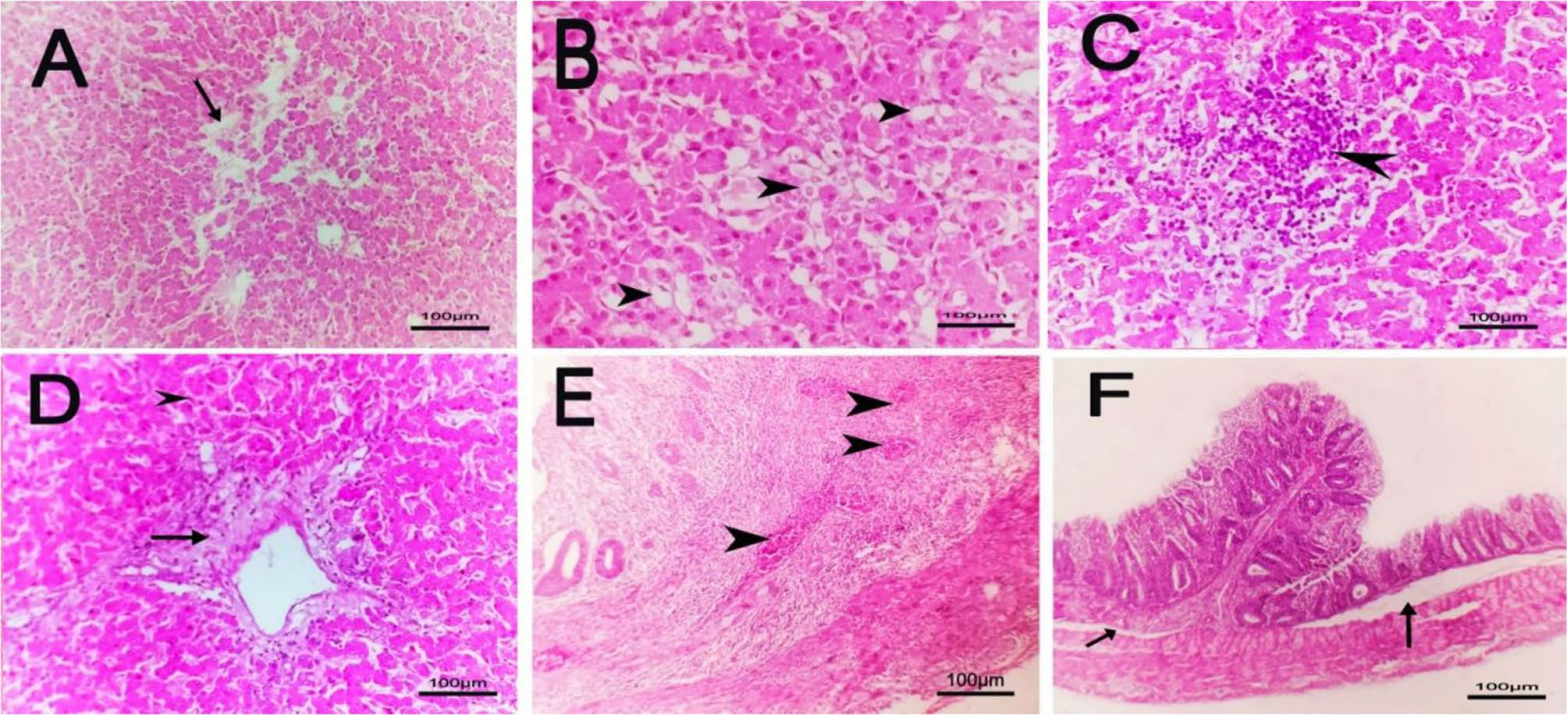
Histopathological lesions in liver and cecal tissue for the treated chicks after the challenge (5th group) with H&E (hematoxylin and eosin), 100×. **A**: liver with focal edema and atrophied hepatocytes. **B**: Liver with diffuse vacuolation of hepatocytes. **C**: Liver with focal mononuclear cells infiltration. **D**: Liver with perivascular coagulative necrosis and pyknotic nuclei. **E**: Cecum with diffuse congestion in submucosal blood vessel. **F**: Cecum with edema under submucosa

Group 5 (challenged with S. Kentucky and then treated with bacteriophage cocktail) exhibited focal edema with atrophied hepatocytes, diffuse vacuolation of hepatocytes, focal mononuclear cell infiltration and perivascular coagulative necrosis with pyknotic nuclei in the liver. The cecum showed diffuse congestion of submucosal blood vessels and edema beneath the submucosa (Fig. 9).

## Discussion

*Salmonella* is one of the major causes of foodborne illness globally. Chickens are considered to be the main reservoir for this zoonotic bacterium [28]. It causes severe illness in chickens, which impedes the development of the poultry industry [2]. In the present study, *Salmonella* was isolated from 18% of diseased chickens collected from farms in Dakahlia Governorate, Egypt. Several studies have been conducted by Shen et al. [3] and Abd El-Mohsen et al. [31], who revealed that the prevalence rates of *Salmonella* species in chickens were 5.66%, 11.36%, 15.5% and 36.54%, respectively. The variations in *Salmonella* isolation may be attributed to risk factors such as flock size, the age of the birds, climatic conditions, season, biosecurity measures and the site of the examined farms. Therefore, the risk factors like poultry house type, flock age, and flock size which effect on the level of *Salmonella* contamination must be investigated.

According to the serotyping results in the present study, 8 *Salmonella* serotypes (*S.* Kentucky, *S.* Typhimrium, *S.* Enteritidis, *S.* Infantis, *S.* Virchow, *S.* Rechovot, *S.* Papuana and *S.* Labadi) were detected, and the most predominant serotype in this study was *S.* Kentucky. Our results agreed to some extent with those of Arkali et al. [32], who reported *S.* Infantis, *S.* Enteritidis and *S.* Typhimurium in chickens in eastern Turkey. Abd El-Mohsen et al. [31] isolated *S.* Kentucky, *S.* Enteritidis, *S.* Typhimurium, *S.* Molade, *S.* Inganda, *S.* Papuana, *S.* Wingrove and *S.* Larochelle from chickens in Assiut, Egypt. There are differences in *Salmonella* serovars between different countries and in different locations in the same country [33]. *Salmonella* Kentucky is frequently isolated from both poultry and humans. It is the principal serovar identified in chickens in the United States [34]. Also, it has emerged as a global zoonotic pathogen [35] and it is among the most common *Salmonella* serotypes associated with poultry worldwide in recent years [36].

Regarding the obtained disc diffusion results, the highest resistance was 94.4% for amoxicillin, followed by 83.3% for ampicillin/sulbactam. The majority of the isolates exhibited multidrug resistance. Our results differ from those of Das et al. [4], who reported resistance to ampicillin and tetracycline of 98.8% and 94.2%, respectively. The resistance or sensitivity of *Salmonella* isolates to antibiotics may be attributed to different levels of flocks’ exposure to antibiotics. The resistance of nearly all of the antimicrobial agents tested in this study necessitates the using of alternatives biocontrol measures with the application of biosecurity measures [37].

In the current study, bacteriophages infecting multidrug-resistant *S.* Kentucky, *S.* Enteritidis and *S.* Typhimurium were isolated from sewage samples collected from different poultry farms and confirmed by the formation of lytic spots and plaques (clearance zone) via spot and plaque techniques in addition to characterization via transmission electron microscopy. Three different Caudovirales phages were identified in this study according to their morphological characteristics which belong to *Autographiviridae*, *Straboviridae* and *Drexlerviridae*. Our results agreed strongly with the results obtained by Bardina et al. [28]. The apparent high prevalence of free-living phages infecting *Salmonella* in poultry farm environment suggested an important role of phages in the ecology and distribution of *Salmonella.* Interestingly, the finding of the present study suggested that the most predominant lytic phages infecting *Salmonella* belonged to *Myoviridae* followed by *Siphoviridae* and *Podoviridae* which is similar to the previous report [38].

With respect to the lyophilization of the recorded bacteriophage cocktail related to the Caudovirales order, the stability, viability and morphological characterization after reconstitution were checked, and the obtained results revealed that the bacteriophages remained stable and viable. Lytic activity was not affected after lyophilization, and no detectable damage to the lyophilized bacteriophage structure was detected. Our findings were supported by those of Merabishvili et al. [39] and Manohar and Ramesh [15]. Gelatin is a good stabilizer, and it can form polymers that support the maintenance of phage morphology, stabilize phage titers during lyophilization and maintain viability after lyophilization for up to 20 months at 4 °C [15]. In the present study, 1% gelatin was used as a stabilizer to maintain the activity of bacteriophages. The active concentrations before and after lyophilization were 1 × 10^10^ and 9 × 10^9^ PFU/ml, respectively. On the other hand, Merabishvili et al. [39] demonstrated the greater ability of sucrose and trehalose, which are used as stabilizers in the lyophilization process, to maintain the phage for a 27-month storage period with a reduction in phage concentration by 1 log.

In the present study, an in vivo experimental model was used to evaluate the ability of a lyophilized bacteriophage cocktail to control multidrug-resistant *S.* Kentucky. Notably, no clinical signs were reported in negative control, group 3 or group 4. The observed clinical signs and PM lesions in group 5 were mild diarrhea, slight enlargement of the cecum and a congested liver. However, severe clinical signs with PM lesions were observed only positive control. No mortalities were recorded in the treated groups with bacteriophage, while group 2 had 30% mortality. It was observed in this study that treatment with a bacteriophage cocktail before challenge with *S.* Kentucky had the ability to reduce clinical signs compared with treatment with a bacteriophage cocktail after challenge (group 5). Our findings agreed with those of Lim et al. [40]. Nabil et al. [26] reported that bacteriophage treatment for several doses in chicks challenged with *S.* Enteritidis and *S.* Typhimurium decreased the number of clinical signs, which disappeared gradually and prevented mortality. In contrast, Ngu et al. [41] reported a decrease in mortality to 11.1% in chickens treated with bacteriophage as a single phage or cocktail phage.

Concerning the recorded growth performance, the parameters improved in the treated groups compared with those in the positive control group. The interpretation of these findings has been previously explained by Sarrami et al. [9], who mentioned that bacteriophage application in broilers may be an alternative to growth-promoting antibiotics because it helps to alter the gastrointestinal tract microbiota and improve the gut morphology and performance parameters including body weight and FCR.

Bacteriologically, *S.* Kentucky was not detected in the cecum, liver or spleen of the bacteriophage-treated groups, and these findings were confirmed in some selected cecal samples using real‒time PCR, which is an accurate and sensitive technique. Our findings were attributed to the ability of bacteriophages to modulate the microbiome of the chicken intestinal tract and reduce pathogenic microbial populations [12]. Additionally, Sorour et al. [42] showed the effectiveness of bacteriophages in reducing *S.* Kentucky colonization in broiler chickens.

Based on bacteriophage counts in the cecal contents (PFU/gm) determined via plaque assays, the mean counts of groups 3, 4 and 5 were 8.6 × 10^7^, 4.39 × 10^8^ and 1.69 × 10^8^ PFU/gm, respectively. The mean bacteriophage content in the challenged and treated groups 4 and 5 was greater than that in the treated and nonchallenged group 3. These results showed the ability of the bacteriophage cocktail to prevent *S*. Kentucky colonization in the cecum and decrease the *S*. Kentucky concentration from 10^5^ to 10^2^ CFU/gm. These results were supported by Toro et al. [43], who reisolated phages from the feces of chickens treated with a cocktail that passed through the digestive tract and was not inactivated and replicated successfully. Overall, treatment with a bacteriophage cocktail at 6 doses prevented *S.* Kentucky colonization before infection, but it reduced *S.* Kentucky colonization after infection. Li et al. [44] reported that treatment with a phage cocktail orally 24 h before or alongside a challenge with *Salmonella* significantly reduced its colonization of the intestinal tract of chickens. Phage application did not negatively affect lymphocyte number or activity, which is important for normal immune system function [45]. In this study, the serum levels of IgM and IgA antibodies were significantly greater in the bacteriophage cocktail-treated groups than in the control group. Our results were supported by those of Sarrami et al. [9], who reported that bacteriophage application in broilers enhances immunological responses by increasing the serum concentrations of immunoglobulin IgM and IgG. Obviously, the levels of IL-4 in the treated group with bacteriophage and not challenged with *S.* Kentucky were greater than those in the treated groups with bacteriophage and infected with *S.* Kentucky. Phage cocktail therapy led to increased levels of IL-4 and IL-10 cytokines that exert anti-inflammatory effects [45].

The histopathological findings in this study revealed normal liver and normal mucosa, muscularis, submucosa and serosa in the cecum of the mice in the groups treated with bacteriophage, while in the other groups, there were different histopathological changes. The findings of this study were supported by those of Cao et al. [46], who reported that phage treatment improved pathological changes and damage to the liver, intestine, and heart in chickens challenged with *Salmonella*. Furthermore, an experimental model designed by Huang et al. [10] demonstrated that the livers of chicks in the control group that received phage showed no obvious pathological changes.

Our experiment obtained preliminary data. The genetic characterization of the phage and experimentally verified data regarding phage safety in relation to the lytic cycle and the absence of any toxin genes should be completed.

## Conclusion

Phage therapy is one of the promising therapeutic alternatives to combat the problem of bacterial resistance to the most available antibiotics. Phages in suspension can undergo physical and chemical stresses such as pH/temperature changes and agitation which can lead to phage loss. This study proved that lyophilization of phage improves the viability and the stability of it for long-term storage.

## Electronic supplementary material

Below is the link to the electronic supplementary material.

Supplementary Material 1

## Data Availability

All data generated or analyzed during this study are included in this published article and its supplementary information files.
