# Supplementary material for: Evaluation of lyophilized bacteriophage cocktail efficiency against multidrug-resistant Salmonella in broiler chickens
Source: BMC Microbiol. 2024 Sep 11;24:338. doi: 10.1186/s12866-024-03467-2 (PMC11389103; doi:10.1186/s12866-024-03467-2)
Supplement: Supplementary file 1 — Supplementary Material 1 [file 12866_2024_3467_MOESM1_ESM.docx]

**Supplementary table (1) Oligonucleotide primers and probes**

| **Gene** | **Primer/ probe sequence (5'-3')** | **Reference** |
| --- | --- | --- |
| *inv*A | GCGTTCTGAACCTTTGGTAATAA | **[25]** |
|  | CGTTCGGGCAATTCGTTA |  |
|  | 5′-FAM-TGGCGGTGGGTTTTGTTGTCTTCT-TAMRA-3′ |  |

**Daum, L.T., Barnes, W.J., McAvin, J.C., Neidert, M.S., Cooper, L.A., Huff, W.B., Gaul, L., Riggins, W.S., Morris, S., Salmen, A. and Lohman, K.L., 2002.** Real-Time PCR Detection of Salmonella in Suspect Foods from a Gastroenteritis Outbreak in Kerr County, Texas. J ClinMicrobiol.2002 August; 40(8): 3050–3052.
